# Potentially Identifying Variables Reported in 100 Qualitative Health Research Articles: Implications for Data Sharing and Secondary Analysis

**Published:** 2023-05

**Authors:** Annie B. Friedrich, Jessica Mozersky, James M. DuBois

**Affiliations:** Medical College of Wisconsin, 8701 Watertown Plank Rd, Milwaukee, WI 53226, USA; Bioethics Research Center, Washington University School of Medicine, 4523 Clayton Avenue, Campus Box 8005, St. Louis, MO 63110-1093, USA; Bioethics Research Center, Washington University School of Medicine, 4523 Clayton Avenue, Campus Box 8005, St. Louis, MO 63110-1093, USA

**Keywords:** qualitative data sharing, health sciences, secondary analysis, potentially Identifying variables, de-identification

## Abstract

There is a growing trend in social science research to share qualitative data in a repository for others to access. However, some researchers are reticent to share qualitative data. One major concern is how to de-identify qualitative data while maintaining adequate contextual detail to allow secondary users to meaningfully interpret de-identified data. To help inform discussions regarding qualitative data sharing, we reviewed 100 qualitative health science studies to determine what potentially identifying variables (PIVs) are reported in the published literature. There are relatively few PIVs reported in each qualitative study; the majority of studies (n=64) reported two or fewer PIVs. The most commonly reported PIVs were profession, sex or gender, and age. Our findings can help guide de-identification efforts going forward as presumably the PIVs that are most commonly reported provide essential contextual details that will also be needed by secondary users, while PIVs that are rarely reported may not provide essential contextual information for interpretation of data. This suggests it is possible to share qualitative data that are both de-identified and useful for secondary analysis. As data are shared, we recommend researchers mask study sites, as these may uniquely increase the chance of re-identification.

## 1. Introduction

In some countries, such as the UK, Australia, and Germany, there has been a growing trend in social science research to share qualitative data in a repository for others to access (ALEXANDER et al., 2019; BISHOP & KUULA-LUUMI, 2017; CORTI, 2000; DuBOIS, STRAIT & WALSH, 2018; KARCHER, KIRILOVA, PAGÉ & WEBER, 2021; LEVENSTEIN & LYLE, 2018). This trend builds on the shift towards open science and the increasing requirements of funding institutions, professional associations, and journals to share and reuse data (ELMAN, KAPISZEWSKI & KIRILOVA, 2014; FELDMAN & SHAW, 2019; NOSEK et al., 2015; TAICHMAN et al., 2016). In the United States (US), quantitative data are frequently shared but the same is not true of qualitative data, which are rarely shared. In a recent survey of over 400 qualitative health researchers in the US, we found that 96% had never shared qualitative data in a repository (MOZERSKY et al., 2021). [1]

The National Institutes of Health (NIH), the largest federal funder of health research in the US, issued a new data sharing policy mandating data sharing as of 2023 regardless of whether data are qualitative or quantitative, including data that are not used in final publications (Notice Identification: NOT-OD-21–013). The policy requires all NIH funded investigators to submit data management and sharing plans to “integrate data sharing into the routine conduct of research” (NATIONAL INSTITUTES OF HEALTH, 2020, n.p.). Qualitative researchers who work in the health sciences, and are grant funded by the NIH, will need to be prepared to share data going forward to meet these new requirements. [2]

At the same time, researchers have expressed multiple concerns regarding qualitative data sharing (BROOM, CHESIRE & EMMISON, 2009; KIRILOVA & KARCHER, 2017; KUULA, 2011; MOZERSKY et al., 2021; MOZERSKY, WALSH et al., 2020; YARDLEY, WATTS, PEARSON & RICHARDSON, 2014). One of qualitative researchers’ primary concerns is that the very process of de-identification would remove contextual details needed by secondary users to meaningfully analyze and interpret the data (BISHOP, 2009; HAMMERSLEY, 2010; MANNHEIMER, PIENTA, KIRILOVA, ELMAN & WUTICH, 2019; MOZERSKY, WALSH et al., 2020). These concerns are reflected in our survey of over 400 qualitative health researchers, where 79% of researchers reported concerns that data cannot be adequately de-identified and 74% said that data sharing would create the potential for misinterpretation of the data (MOZERSKY, WALSH et al., 2020). [3]

Qualitative data are often rich in detail and context not captured by quantitative methods, and frequently explore sensitive or stigmatized topics, especially in relation to health (BARBOUR, 2000; BROOM et al., 2009; DICKSON-SWIFT, JAMES, KIPPEN & LIAMPUTTONG, 2006; GUILLEMIN & HEGGEN, 2009). The ability to capture detailed, subtle, and often hidden aspects of people’s experiences related to health is what makes qualitative methods a rich and important addition to quantitative data. However, the non-numeric nature of qualitative data also creates de-identification challenges. Removing standard variables such as names, addresses, or ID numbers may not suffice when data contain uniquely identifying information based on combinations of information such as job title, organization, and year (e.g., CEO of Google in 2006) or a sufficient combination of contextual details that may enable re-identification (e.g., a male, psychiatric nurse at local hospital X) to infer an identify (DuBOIS et al., 2018; KIRILOVA & KARCHER, 2017). [4]

However, we currently lack clear guidance on how to adequately de-identify qualitative data in a way that maintains confidentiality while leaving sufficient contextual detail to enable secondary use. In the US, the Health Insurance Portability and Accountability Act (HIPAA) provides a list of 18 “safe harbor” identifiers, including names, birth dates, medical record or device numbers, and phone numbers that must be removed for data to be considered de-identified. However, HIPAA requirements were designed for quantitative data found in the electronic medical record, and the majority of the 18 HIPAA safe harbor identifiers are unlikely to be present in qualitative data at all (GUPTA et al., 2021). HIPAA identifiers are specific to the US regulatory context and removing them helps to meet US regulatory de-identification requirements, but this alone is not sufficient for de-identifying qualitative data. Data repositories such as the UK Data Archive distinguish between direct and indirect identifiers (UK DATA SERVICE, 2023). Indirect identifiers—like a unique profession—present greater challenges when de-identifying qualitative data as they may be identifiable when combined with other data. Importantly, there are no agreed upon standards for determining when qualitative data can be considered de-identified or what variables must be removed to ensure data cannot be re-identified, or by whom (as it may always be that the interviewee and interviewer will be able to identify themselves). [5]

Qualitative data are much more likely to contain other types of variables that would not be considered HIPAA identifiers but that could potentially identify participants when combined with one another (McLELLAN, MacQUEEN & NEIDIG, 2016; TOLICH, 2004; TSAI et al., 2016). Throughout this paper, we refer to these as potentially identifying variables (PIV) because on its own a PIV will not identify a participant, but when multiple PIVs are combined, re-identification becomes more likely. [6]

In order to ethically and responsibly share qualitative data, researchers must balance the need to remove enough details to protect individual identities while leaving as much contextual details as possible to enable secondary users to interpret the data (MOZERSKY, WALSH et al., 2020). Data that are stripped of too many details may protect individual identities, but if the remaining data are rendered useless for secondary analyses because too many details have been removed, then the goals of data sharing cannot be met. [7]

For some qualitative researchers, the very notion of sharing data for others to use is problematic because, they argue, only the original investigators are capable of interpreting the data correctly due to their specific contextual knowledge of the data (FELDMAN & SHAW, 2019; KUULA, 2011; MOZERSKY, WALSH et al., 2020). In fact, some researchers only allow secondary use of their qualitative data when it will involve their direct collaboration with secondary researchers (FELDMAN & SHAW, 2019). From some methodological standpoints, researchers understand qualitative data as jointly constructed or “generated” by researcher and respondent in an iterative and intersubjective process that cannot be replicated by secondary users (BROOM et al., 2009, p.1164). [8]

Despite the concerns regarding secondary use, there are numerous benefits of qualitative data sharing such as enabling new research with existing data in cost-effective ways, collating existing data, training students in qualitative analysis with original data sets, and enhancing transparency in the research enterprise (ANTES, WALSH, STRAIT, HUDSON-VITALE & DuBOIS, 2018; BISHOP, 2009, 2014; CARUSI & JIROTKA, 2009; CHATFIELD, 2020; CORTI, 2007). Qualitative research participants may also support data sharing especially if it saves resources or avoids the need for recontact. We conducted qualitative interviews with 30 research participants who had taken part in sensitive qualitative health studies and found that the majority supported data sharing with other researchers so long as data were de-identified (MOZERSKY, PARSONS et al., 2020; VANDEVUSSE, MUELLER & KARCHER, 2022). Qualitative data sharing may also help mitigate study participant “research fatigue,” or the psychological and emotional exhaustion that study participants, particularly those from small and/or marginalized communities, may experience when asked to participate in countless research projects. With increased qualitative data sharing, researchers can access important data sets without unduly burdening research participants (ASHLEY, 2020). At the same time, it is important to ensure that researchers do not become overly reliant on a single shared data set to avoid entrenchment. Realizing the benefits of data sharing requires overcoming the barriers and concerns of qualitative health researchers. [9]

In this paper, we set out to determine what potentially identifying variables (PIV) are reported by qualitative researchers in 100 qualitative health sciences articles that employed a focus group or interview methodology during a recent 12-month period. Given that a major barrier to sharing qualitative data is the claim that de-identification will remove too many contextual details and make secondary analyses unfeasible, we sought to examine what variables are actually reported in these articles. Our analysis is premised on the assumption that the variables reported by researchers in published literature are the essential contextual details needed for interpreting the data, and therefore provide the minimum adequate contextual details needed by secondary users. If a variable is not reported, then we assume it was not essential for interpretation of the data. Our analysis of these articles provides information on the number, types, and ways in which variables are commonly reported in qualitative health science research. This information can help inform future efforts to determine which identifiers are needed for interpretation by secondary users, and which can—in the view of authors—be shared without risking re-identification of participants. [10]

The data reported here were gathered as part of a larger NIH-funded project [RO1HG009351–04] to overcome barriers and facilitate ethical and responsible qualitative data sharing. This includes developing guidelines and software to support health science researchers in de-identifying qualitative data prior to sharing with a repository where others can access them for secondary use (DuBOIS et al., 2018). In our project, we analyzed data from interviews or focus groups—primarily transcripts from recorded conversations. We do not currently intend to share other forms of qualitative data such as field notes or visual data such as photographs, as these present greater challenges for de-identification. [11]

We begin with a description of our methods, including how we determined what could be considered a potentially identifying variable (Section 2). We report the results of our analysis in Section 3 and discuss implications for data sharing and secondary analysis in Section 4. [12]

## 2. Methods

### 2.1 Article selection

To collect a broad sample of qualitative health science research involving interviews or focus groups, we searched PubMed for articles published between November 1, 2019 and October 31, 2020 using the search terms: “qualitative AND (“interview” OR “focus group”). We randomly selected 50 articles that met our inclusion criteria (human subjects research, published in English, indexed in MEDLINE, using an interview or focus group methodology). We also randomly selected 50 qualitative health science research articles from top-ranked health science journals as indicated by impact factor. This strategy ensured that we captured a wide variety of qualitative health science articles that could serve to represent the field. Our full search strategy for collecting a representative sample of qualitative health science research has been described elsewhere (MOZERSKY, FRIEDRICH & DuBOIS, 2022).^1^ [13]

### 2.2 Determining what potentially identifying variables are reported

All authors were involved in initial codebook development, which was originally developed as part of our larger project to determine what non-HIPAA identifiers were likely to be present in qualitative data. These data informed the development of software we are creating that assists with de-identification of qualitative data (GUPTA et al., 2021). Our codebook contained the 18 HIPAA identifiers, and a list of other potentially identifying variables that may be reported in qualitative health science research (ibid.) (see Table 1). We allowed for inductive coding if we found any PIVs during coding that we did not anticipate and that were not contained in our original codebook. [14]

We differentiate between PIVs that are inclusion criteria and apply to the entire sample (e.g., nurses, students) from those PIVs that are connected to a specific participant quote (e.g., 35-year-old female). Both could potentially be identifying of an individual participant, but this will almost always be due to a combination of PIVs, rather than a single PIV. [15]

### 2.3 Determining how potentially identifying variables are reported

We also coded for any indication that researchers attempted to mask a particular PIV to protect participant privacy. We created a “masking” code which was defined as any attempt to present data more broadly or mask an inclusion or individual identifier, including generalization (e.g., health center in an urban area), obvious redaction, using study ID numbers to identify participants, or using pseudonyms. [16]

### 2.4 Characterizing study site

We also coded each article for whether the research was carried out at a single site or institution, multiple sites or institutions, or more broadly within a particular community or group of participants. We included study site because knowing the site may increase the chances that an individual could be re-identified when combined with other PIVs, especially if it is the only site where research was conducted. We distinguished study site from an organization or institution by defining study site as the location where the specific study took place, whereas an organization or institution could be mentioned anywhere in the data but did not necessarily refer to the specific site where the research was conducted. [17]

During the first phase of coding, two coders (AF and JM) blind coded two articles, discussed and resolved differences in coding, and made changes to the codebook. The coders repeated this process until codes were consistently applied to the articles without major discrepancies between the raters. The primary coder (AF) proceeded to code half of the articles, at which point another blind coding check was conducted with the second coder (JM) to ensure ongoing agreement and consistent application of codes. Coders resolved differences in coding during consensus meetings and revised the codebook accordingly.

## 3. Results

### 3.1 What potentially identifying variables are reported?

We first assessed which PIVs were reported in the articles and whether these characteristics applied to all study participants (inclusion PIV) or were used to provide key contextual or individual participant characteristics immediately following a participant quote (individual PIV) (Table 2). Given the unique nature of study site as a potentially identifying variable, we analyzed this PIV separately (see Table 5). [19]

The most commonly reported PIV was profession (reported in 64 articles); in over half of these instances (36 articles) profession was an inclusion criterion (e.g., a study of nurses or residents). The remaining 28 articles reported profession in relation to individual participants following a quote, and this was masked in 16 articles. Sex or gender (45 articles) and age (23 articles) were the next most commonly reported PIVs (Table 2). Sex or gender was never masked and was most often reported as an individual PIV following a quote. Age was never an inclusion PIV, and very rarely masked as an age range (3 out of 23 studies). The remaining PIVs—institution or organization names (not related to study site), race, ethnicity, indigenous status or nationality, marital status, education level, and rare disease or illness—were rarely reported, and we saw no instances of LGBTQI or foreign language or dialect PIVs. [20]

Of the three most commonly occurring PIVs (profession, sex/gender, and age), age and sex/gender are reported together in 14 articles, profession and sex/gender are reported together in 21 articles, and age and profession are reported together in 22 articles. Only 14 articles report all three of the most commonly occurring PIVs in combination.

### 3.2 How are potentially identifying variables reported?

We also assessed whether researchers attempted to mask a particular PIV to protect participant privacy. Eighty-six articles utilized at least one masking strategy, and the most common masking practice was generalization, with almost 70% of articles employing this masking strategy for an inclusion PIV, individual PIV, or study site. Assigning a participant ID was another common masking strategy. Using a pseudonym or fully redacting information were rarely utilized (Table 3).

### 3.3 How many PIVs are reported in each study?

**Figure 1:**
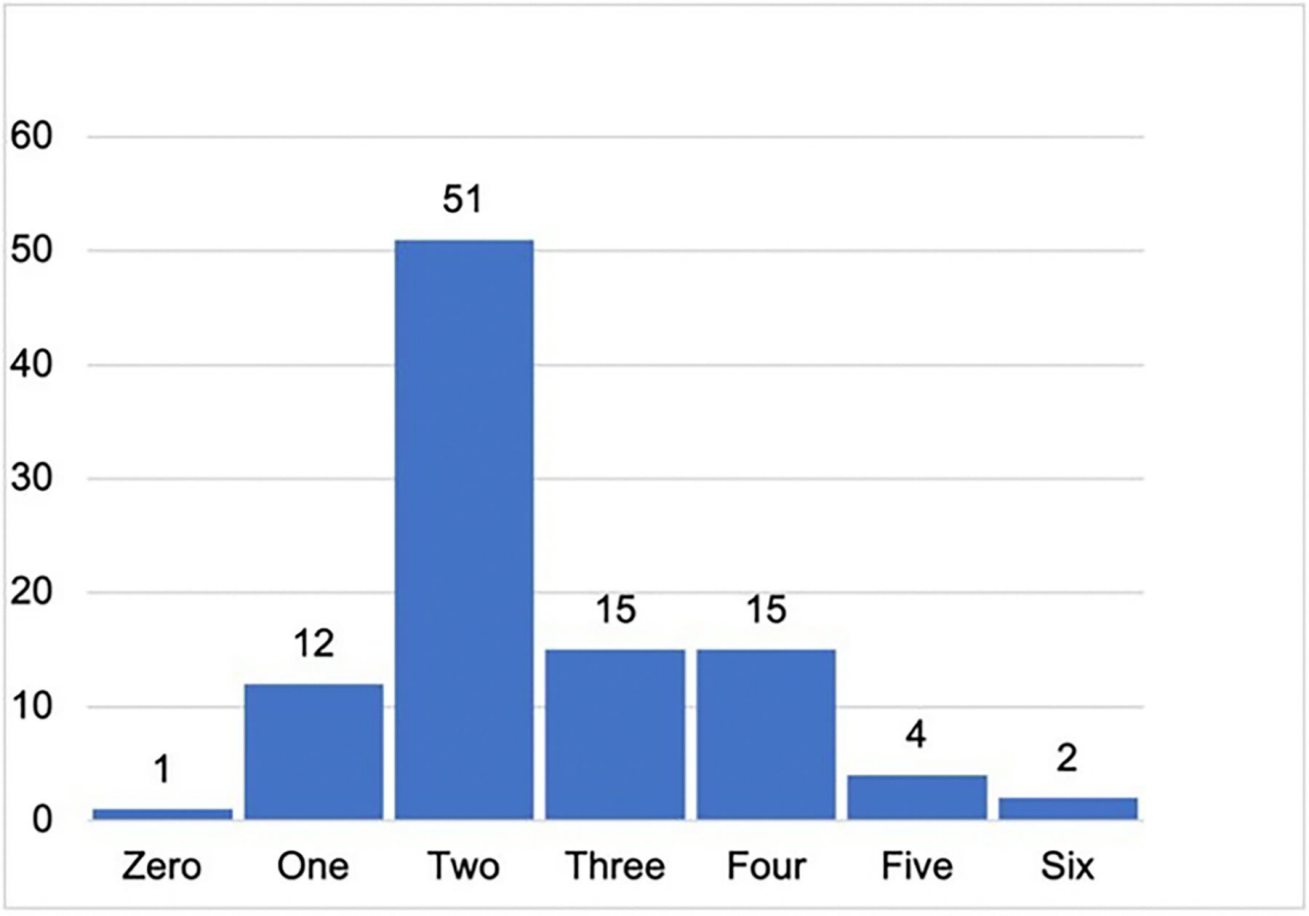
Percent of articles with zero to six PIVs (inclusion + individual)

We calculated how many PIVs were reported within each article, distinguishing between inclusion PIVs and individual PIVs. In forty articles, researchers reported no individual PIVs (Table 4). In the majority of articles (64), researchers reported 2 or fewer PIVs of any kind (either inclusion or individual PIVs) (Figure 1).

### 3.4 Characterizing study site(s)

Because knowing an individual study site could be highly identifying for participants in certain contexts, especially in combination with other reported PIVs, we analyzed whether the study was conducted at a single site or institution, multiple sites or institutions, or conducted more broadly within a community. We also assessed whether any masking strategies were employed. Thirty-two studies were conducted at a single site, and 13 of these studies named the specific institution where research was conducted. Twenty-two studies were conducted at multiple sites, and only 1 study named the specific participating institutions. Forty-six studies were conducted within broader communities or with a generalized group of participants, rather than a particular site or institution. Generalized or broad community research may be masked by the nature of the research (i.e., there is no particular institution or organization to pseudonymize or generalize) (Table 5). We also assessed whether any HIPAA geographic locations (geographic subdivisions smaller than a state) were reported. In thirty-nine studies, researchers included a HIPAA geographic location to further describe study site. We found 4 articles describing broad community research in which participants were individually identified as belonging to one of the specific communities where the research was conducted, potentially increasing their identifiability.

## 4. Discussion

One main argument against qualitative data sharing is that de-identification to adequately protect participant anonymity will remove important contextual details needed for accurate secondary interpretation of the data (BROOM et al., 2009; FELDMAN & SHAW, 2019). In light of the increasing trends and requirements for qualitative data sharing, we sought to determine what potentially identifying variables (PIVs) are reported in a representative sample of qualitative health science literature. This information can help guide de-identification efforts going forward by providing a better understanding of which PIVs are most commonly reported and presumably considered to be essential contextual details needed by secondary users, and which PIVs are rarely reported and presumably not viewed as essential contextual information for interpretation of data. To our knowledge, this is the first study to seek this information. [25]

Our findings show that there are relatively few PIVs reported in individual published qualitative health science research studies; most variables we anticipated finding based on our codebook are not commonly reported by qualitative researchers. We found no instances of the 18 HIPAA safe harbor identifiers apart from cities describing study site, which is unsurprising since most identifiers were designed with quantitative data in mind (GUPTA et al., 2021). More notably, the majority of non-HIPAA PIV categories in our codebook were also rarely, or never, reported (i.e., race or ethnicity, education, rare diseases, LGBTQI status). The most commonly reported PIVs were profession, sex or gender, and age. All three of the most commonly occurring PIVs were reported together in only 14 articles. Because PIVs or study sites may be masked, these combinations likely provide important contextual information while maintaining a low risk of participant re-identification. This suggests that authors likely can report age, sex/gender, and profession—alone or in combination—to further contextualize their data. [26]

In the majority of studies (n=64), researchers reported two or fewer PIV categories, and in nearly half of the studies (n=40), researchers reported no individual PIVs following a participant quote at all. In these cases, the only PIVs were inclusion criteria that applied to all participants (i.e., females, physicians, etc.). This suggests that many variables could be removed from data for the purpose of de-identification while leaving the most essential variables for context and interpretation in the data. Of course, when such information will not enable inferential re-identification of participants, it may be safely left, which would provide secondary users with more natural and complete text and associated context. [27]

We also analyzed study site information because knowing the study site may increase the chances that an individual could be re-identified when combined with other PIVs. The majority of studies (n=68) were conducted at multiple sites or were generalized to broad communities or participant groups, which reduces the chance of re-identification based on a specific location. However, we did find a small number of generalized and broad community articles in which researchers individually identified participants as belonging to one of the specific communities or cities where the research was conducted, potentially increasing their identifiability. Thirty-two studies took place at a single study site, 13 of which named specific institutions. These single-site studies may pose the greatest risk of reidentification of participants when combined with other PIVs, particularly individual PIVs attached to participant quotes. For example, the following participant quote was published in a study that took place at the University of California San Francisco (UCSF):

“[The patient] said ‘I looked at your name and thought I would see someone that looks more like me. I don’t want to talk to you.’ And she started yelling at us to leave. Then the attending came in and she started yelling at him too because he was black. [Fourth-year medical student, woman, Latina]” (WHEELER et al., 2019, p.1680). [28]

Because we know the participant is a female of Hispanic descent (referred to as “Latina” by the authors) and a fourth-year medical student at a particular institution (UCSF), colleagues may potentially be more able to re-identify her. However, because UCSF is a large institution with many Hispanic students, study authors likely felt that the risk was low. In contrast, it is much harder to identify participants in multi-site studies or when participants are drawn from a broader region or country who meet study criteria (TSAI et al., 2016). Using the example quote above, if the study was conducted at 3 academic medical centers, the chances that this individual could be re-identified in combination with other PIVs is greatly reduced. Researchers will need to weigh the risks associated with conducting their study at a single site and take steps to mitigate those risks, such as appropriate masking or generalization to include meaningful context about their site while also protecting participants. Researchers will also need further education, given that 13 out of 23 single site studies did not mask the site, while the majority of multi-site studies were masked or generalized in some way (21 out of 22 studies). [29]

Our overall findings suggest that published data do not include many potentially identifying contextual details. Little violence would be done to shared data by masking some PIVs; and some PIVs (profession, age, and sex/gender) can, it appears from these 100 studies, be safely shared unmasked without a significant risk of identifying participants. At the same time, many questions remain about how to conduct responsible and ethical secondary analysis of qualitative health science research. Currently, de-identification of qualitative data is primarily done manually with researchers removing what they deem to be necessary based on the context, and we lack guidelines and standards for how to safely and ethically de-identifying qualitative data (KIRILOVA & KARCHER, 2017). Given the nature of qualitative research, there may be no definitive combination or number of identifiers that pose a particular risk to participant privacy, so individual researchers will need to assess each study individually to determine what PIVs should be removed. We found that participants are supportive of sharing their qualitative data so long as it is de-identified, but questions remain about when qualitative data can be considered adequately de-identified, which requires balancing removing identifying information while leaving enough contextual detail to enable secondary use (MOZERSKY, PARSONS et al., 2020). Study participants should also be given the opportunity to share their perspectives on what information they consider identifying, which is an area for future research. [30]

We also lack guidelines on appropriate secondary use, which is an important area for future research (KIRILOVA & KARCHER, 2017; YOON, 2014). Data repositories and curators with experience with qualitative data can work with researchers to ensure that they provide appropriate context for the data and document what the data do not contain—to help secondary users assess how the information available to them differs from that available to the original researchers, and help convey the researchers’ original epistemological or theoretical commitments (KARCHER et al., 2021). While some critics argue that it is not possible to reuse archived data without access to the original context in which it was produced, others argue that “recontextualizing data” opens up new possibilities for data reuse and theory building (BISHOP, 2006; MOORE, 2006). In fact, researchers’ intimate knowledge of the original research context can be a boon to sharing, as they are well positioned to make necessary modifications to data without harming participants or the data themselves (KIRILOVA & KARCHER, 2017). [31]

Researchers can also choose to make their qualitative data only available via restricted access, which requires that secondary users have the appropriate approvals prior to accessing the data. Whenever qualitative data is archived for reuse, sharing and reanalysis should be carried out in ways that fit the particular research tradition (KAPISZEWSKI & KIRILOVA, 2014; LUPIA & ELMAN, 2013). Some data collection methods such as ethnography with extensive personal field notes may create greater challenges for de-identification prior to sharing. Researchers, data curators, and academic librarians can work together to find the balance between sharing as much as possible without introducing undue additional risk (KIRILOVA & KARCHER, 2017). Restricted access is likely the most appropriate way to share qualitative data given the lack of guidance regarding de-identification and secondary analyses currently. Regardless of how qualitative data are shared, it is essential that researchers obtain permission for data sharing from the research participants themselves. Consent forms must convey information regarding data sharing in a way that is understandable and acceptable to participants. [32]

Our study has several important limitations. First, we focused only on qualitative health science research, which does not represent the breadth and diversity of contexts where qualitative research is conducted. Similarly, our focus on studies involving interviews and focus groups cannot be generalized to other methods such as participant observation or oral histories. In fact, oral histories involve making participant names public; de-identification concerns are unlikely to be relevant for oral histories, while field notes from participant observation may be especially challenging to de-identify and may not be suitable for sharing. Yet even with these limitations, our findings contribute to important ongoing debates and discussion about secondary use and suggest that qualitative health science researchers may be able to share interview and focus group data much more readily than critics claim. [33]

## Figures and Tables

**Table 1:** Categories of potentially identifying variables reported in the sample of articles

Identifier	Description
HIPAA safe harbor	
HIPAA safe harbor	Any of the 18 HIPAA safe harbor identifiers: names; geographic subdivisions smaller than a state; all elements of dates (except years) for dates that are directly related to an individual (including birth date, admission date, discharge date, death date, and all ages over 89); telephone numbers; vehicle identifiers and serial numbers, including license plate numbers; fax numbers; device identifiers and serial numbers; e-mail addresses; Web Universal Resources Locators (URLs); social security numbers; internet protocol (IP) addresses; medical record numbers; biometric identifiers, including finger and voice prints; health plan beneficiary numbers; full-face photographs and any comparable images; account numbers; certificate/license numbers
Non-HIPAA safe harbor	
Geographic location	Any reference to a geographic area that is not categorized as a HIPAA safe harbor identifier; includes any reference to a country, a US state, or an international equivalent of a state or similar-size region
Institution name/organization	Any reference to the name of an institution or organization
Race, ethnicity, indigenous status, or nationality	Any reference to race or ethnicity, as well as indigenous groups and tribes, and country when used to indicate the nationality of an individual
Age	Any reference to the age or age range of a person that is not categorized as a HIPAA safe harbor direct identifier of age (includes references to age in years, months or weeks but not birth dates which would be considered a HIPAA direct identifier)
*Profession or professional title^1^	Any professions and jobs including titles and qualifications
Rare disease or illness	Any reference to a rare disease that is contained in https://globalgenes.org/rarelist/
LGBTQI/sexual orientation	Any reference to sexual orientation or gender identity that is not heterosexual or cis
*Foreign language or dialect	Any reference to a foreign language or dialect as it pertains to individual participants
*Sex or gender	Includes only explicit references to sex or gender
*Education level	General reference to education level attained by participant (elementary, trade school, etc.)
Non-HIPAA safe harbor	
*Marital status	General reference to marital status of participant (single, widowed, etc.)
*Type of study site: single, multi-site, or generalized/broad community	Single: research took place at one particular institution or organization Multi-site: research took place at two or more particular institutions or organizations Generalized/broad community: research took place within a generalized or broad community setting (participants with a particular disease, nursing students in Australia, etc.)

1 “*”: These identifiers are not included in our current software.

**Table 2:** Number of articles containing potentially identifying variables^1^

Potentially Identifying Variables (PIV)	N=100	Inclusion PIV	Individual PIV	Masked (Individual PIV)^2^
Profession or professional title	64	36	28	16
Sex or gender	45	9	36	0
Age	23	0	23	3 (age range)
Institution or organization names (not related to study site)	8	0	8	5
Race, ethnicity, indigenous status, or nationality	7	2	5	0
Marital status	4	0	4	0
Education level	2	0	2	0
Rare disease or illness	1	1	0	0
LGBTQI/sexual orientation	0	0	0	0
Foreign language or dialect	0	0	0	0

1 We treated inclusion PIV and individual PIV as mutually exclusive.

2 As a general rule, in order to adequately characterize study participants, inclusion PIVs cannot be masked, and we found no instances of masked inclusion PIVs. However, in some studies the study site had been masked. See Table 4 for study site descriptions and masking practices.

**Table 3.** Masking characteristics

Masking	N=86^1^	Example
Generalization	69	“‘Patients do not come for screening because they don’t know the risks and negative consequences of hypertension’ (Participant 5, Health Administrator, Khatlon Oblast)” (CHUKWUMA, GONG, LATYPOVA & FRASER-HURT, 2019, p.8). *Individual PIV: profession generalized as “health administrator*” “Medical professionals involved in the perioperative care of patients undergoing spine surgery at a tertiary academic medical center … were recruited for participation through purposeful sampling*”* (WARNER et al., 2020, p.1907). *Study site: generalized as* “*tertiary academic medical center”*
“Participant,” #, or letter	49	“For example, one focus group participant stated, ‘I live in a really agriculture rich rural area and I’m confident that a lot of our cancer diagnoses, not just for breast cancer but for many, many kinds of cancer, are directly related to the amount of chemicals that are being sprayed’” (OHAYON, NOST, SILK, RAKOFF & BRODY, 2020, p.8). “‘Older nurses are less able to accept new knowledge than younger nurses’ (Nurse 21)” (LI, SUN, WAN, CHEN & SUN, 2020, p.5).
Pseudonym	12	“‘I bet you couldn’t walk in my shoes because all the stuff you got to endure behind these walls of pain. There’s a lot you got to go through … [and] I’ve been doing this for 11 years... people adapt to their surroundings, but to get used to this life, I don’t [think] you can.’ (Michael, a pseudonym, as with all subsequent quotations)” (REITER et al., 2020, p.S59).
Redaction	8	“As one participant stated, ‘You’ve got these major corporations that are paying zillions of dollars to get their cancer-causing products out there, and they’re going to do anything they can to discredit and squash the efforts of anyone who’s trying to rise up against them. And with the funding that they have they can be far more successful than a volunteer advocacy-organization based in rural [state omitted] …’” (OHAYON et al., 2020, p.10).

1 Total is >86 because several masking techniques could be used in one article.

**Table 4:** Number of inclusion and individual PIV categories reported per article

Number of PIV Categories Reported per Article	Articles Reporting Inclusion PIVs	Articles Reporting Individual PIVs
0^1^	1	40
1	55	33
2	41	16
3	3	6
4	0	3
5	0	2
6	0	0
TOTAL # of articles (N=100)	100	100

1 This article reported on individuals connected to multiple historical and international mass unemployment events and thus included no PIVs per our codebook. (DAVIES, HOMOLOVA, GREY & BELLIS, 2019).

**Table 5:** Type of study site

Type of Study Site	N=100	Unmasked	Unmasked Example	Masking
Single	32	13	“We conducted a qualitative investigation based on facilitated focus groups with convenience samples of internal medicine hospitalist physicians, internal medicine residents, and medical students affiliated with the University of California, San Francisco, School of Medicine (UCSF)” (WHEELER et al., 2019, p.1679).	19
Multi-site	22	1	“This mixed-methods study of 3WP [3 Wishes Project] was implemented in ICUs at the following 3 additional locations beyond the original site at St. Joseph’s Healthcare in Hamilton, Ontario: St. Michael’s Hospital in Toronto, Ontario; Ronald Reagan UCLA Medical Center in Los Angeles, California; and Vancouver General Hospital in Vancouver, British Columbia” (VANSTONE et al., 2020, p.1).	21
Generalized/broad community	46	N/A	“We devised a purposive sampling approach to recruit staff currently employed in the United Kingdom (UK) mental health services with adequately diverse characteristics to be broadly representative of the UK mental health workforce” (BAKER, CANVIN & BERZINS, 2019, p.2).	N/A

## Footnotes

1 The articles included in analysis are listed in the Appendix. Please contact James DuBOIS at duboisjm@wustl.edu to request the codebook and code application tables underlying this analysis.
